# Molecular nutrition in life course perspective: Pinpointing metabolic pathways to target during periconception

**DOI:** 10.1111/mcn.13474

**Published:** 2023-02-15

**Authors:** Sergej M. Ostojic, Laszlo Ratgeber, Jozsef Betlehem, Pongrac Acs

**Affiliations:** ^1^ Department of Nutrition and Public Health University of Agder Kristiansand Norway; ^2^ Faculty of Health Sciences University of Pécs Pécs Hungary; ^3^ Applied Bioenergetcis Lab, Faculty of Sport and Physical Education University of Novi Sad Novi Sad Serbia

**Keywords:** creatine, metabolic diseases, nutrition, one‐carbon metabolism, periconception, pro‐inflammatory

## Abstract

Lifecourse nutrition encompasses nourishment from early development into parenthood. From preconception and pregnancy to childhood, late adolescence, and reproductive years, life course nutrition explores links between dietary exposures and health outcomes in current and future generations from a public health perspective, usually addressing lifestyle behaviours, reproductive well‐being and maternal‐child health strategies. However, nutritional factors that play a role in conceiving and sustaining new life might also require a molecular perspective and recognition of critical interactions between specific nutrients and relevant biochemical pathways. The present perspective summarises evidence about the links between diet during periconception and next‐generation health and outlines the main metabolic networks involved in nutritional biology of this sensitive time frame.

## Key messages

- Nutrition can modulate normal periconception and subsequent well‐being, yet we still lack a fundamental understanding of the main metabolic networks involved in nutritional biology of this sensitive time frame.
- Besides one‐carbon metabolism, arguably a key regulatory network affected by periconceptional nutrition, several other nutrition‐related intracellular signals are recognised as important to support normal periconception, including high‐energy phosphate metabolism, gut microbiota‐driven factors, peroxisome proliferator‐activated receptors, adiponectine, mechanistic target of rapamycin and retinol‐binding protein‐4.
- To advance research related to molecular nutrition and periconception, the scientific community must embrace the theranostics approach in research planning and knowledge translation, and monitor additional target molecules and pathways.

## 1 NUTRITION AND PERICONCEPTION: AN OVERVIEW

Periconception could be defined as a 6‐month period in women embracing oocyte growth, fertilisation, conceptus formation and development to Week 10 of gestation (Louis et al., 2008), including a 3‐month preconception period in men for sperm formation and maturation and fertilisation. This is the most sensitive period of human development, and many environmental factors can modulate normal periconception and subsequent well‐being (for a detailed review, see Fazeli & Holt, 2017; and Simeoni et al., 2018). Among others, nutrition in this time window is often considered critical in terms of both short‐ and long‐term health outcomes, with respect to the neurological, cardiometabolic and oncological disorders, from the neonatal period to adulthood (Koletzko et al., 2019; Reijnders et al., 2019). Recent studies investigating maternal western diet's harmful impacts on fetal development in the nonhuman primate model (Friedman, 2018; Wesolowski et al., 2018), as well as dietary recommendations for pregnant women (Marshall et al., 2022), might help set the stage for how metabolic regulators are essential in mediating the effects of maternal diet and fetal supplies on embryonic and fetal development. A vast repercussion of periconception diet is illustrated by a recent study where maternal over‐ or undernutrition during the periconception affected the fertility of the male offspring via long‐lasting metabolic alterations (Zambrano et al., 2021). This suggests that the male reproductive system is developmentally programmed while possible adverse effects of a periconceptional diet could linger to children two generations away. The interconnection between low parental folate intake and neural tube defects or paediatric malignancy was among the first in setting out a scene for a periconceptional nutrition‐for‐children health framework (Rayburn et al., 1996; Swedish Council on Health Technology Assessment, 2007; Toren et al., 1996; Wilson et al., 2003), yet several other critical dietary factors identified via either human or preclinical studies could be associated with ill health in the next generation.

For instance, maternal and paternal obesity before conception can alter the molecular composition of both oocytes and sperm, ultimately increasing the incidence of obesity and metabolic disorders in offspring (Lane et al., 2015), with paternal periconceptional body weight may also affect daughters’ breast cancer risk (Fontelles et al., 2016). Maternal dietary intake of methyl‐group donors (e.g., choline, folate, betaine, methionine) during the periconception period can influence infant metabolism, growth and appetite regulation (Pauwels et al., 2017), with impaired methylation status in parents during periconception also associated with smaller placental growth trajectories (Hoek et al., 2021). Poor parental nutrition during the periconceptional can affect fertility and early development via one‐carbon metabolic pathways that also involve the utilisation of methyl‐group donors (Steegers‐Theunissen et al., 2013). The Rotterdam Periconception Cohort identified weak‐to‐moderate associations between embryonic growth and specific maternal lifestyle factors related to diet, including vegetable and fruit intake, and alcohol consumption (Van Dijk et al., 2018). In line with this, inadequate periconceptional maternal vegetable intake can negatively impact embryo development during the preimplantation period after intracytoplasmic sperm injection treatment (Hoek et al., 2020). Maternal Western dietary patterns (e.g., a diet high in meat, pizza, legumes and potatoes, and low in fruits), accompanied by impaired methylation status, were associated with a higher risk of congenital disabilities (Vujkovic et al., 2007). Periconceptional plant consumption could extend to long‐term health effects, with increased maternal intake of vegetables, fruit and vitamin C that prevent against behavioural problems in pre‐school children (Miyake et al., 2020). A maternal diet rich in proinflammatory foods (such as fried foods, sodas, refined carbohydrates and red meat) is associated with smaller offspring birth sizes and a higher risk of offspring being born small for gestational age (Chen et al., 2021). Maternal vitamin D deficiency is another nutritional element associated with an increased prevalence of heart problems in offspring (Koster et al., 2018), while vitamin D repletion during pregnancy minimises the risk of certain adverse outcomes, such as preterm birth and asthma in children (Wagner & Hollis, 2018). In addition, high maternal vitamin E by diet and supplements is associated with an increased risk of congenital heart defects in offspring (Smedts et al., 2009).

Additional dietary factors linking periconceptional diet and offspring health are identified from animal studies. For example, preclinical data suggest that maternal dietary protein restriction can lead to compensatory fetal growth changes resulting in cardiovascular, metabolic and behavioural diseases in adult offspring (Fleming et al., 2015), as well as poor mineral density (Lanham et al., 2021). On the contrary, chronic metabolic stress and impaired fetal development are seen in females following periconceptional exposure to a high‐protein diet (Mitchell et al., 2009), implying the importance of a balanced protein intake for normal fetal growth. In addition, maternal dietary protein perturbations during conception and early gestation can also alter male testis development and delay puberty (Copping et al., 2018). Maternal supply of omega‐3 polyunsaturated fatty acids can alter mechanisms involved in oocyte and early embryo development, including mitochondrial distribution, calcium homoeostasis and antioxidant‐oxidant status (Wakefield et al., 2008). Interestingly, a methyl‐deficient diet during the periconception period programs glucose homoeostasis in adult male but not female offspring (Maloney et al., 2011), suggesting gender‐specific changes in glucose turnover. Intrauterine vitamin D deficiency predisposes offspring to long‐term adipose tissue consequences and possible adverse metabolic health complications (Belenchia et al., 2017). A few recent reviews provide an extensive compendium of additional studies describing the links between parental nutrition during periconception and next‐generation health (Adair, 2014; Ashworth et al., 2009), and several metabolic pathways might be involved in the relationship. We outlined below the main metabolic networks involved in the nutritional biology of this sensitive time frame.

## 2 ONE‐CARBON METABOLISM

One‐carbon metabolism is a biological network that integrates nutrient status from the environment to yield multiple biological functions (Mentch & Locasale, 2016). This pathway appears to be modulated via various epigenetic mechanisms related to periconception maternal environment, influencing fetal growth and development (Rubini et al., 2021). S‐adenosylmethionine (SAM) is a central player in one‐carbon metabolism, acting as a universal methyl donor for many methylation reactions that involve lipids, DNA and histone proteins. The SAM turnover is influenced by folate‐homocysteine homoeostasis during early embryonic development (Taparia et al., 2007). Several trials suggest that diet‐driven modulation of the folate‐dependent one‐carbon metabolism during periconception could affect fetal and neonatal health (Boyles et al., 2008; Dominguez‐Salas et al., 2013; Liu et al., 2020; Steegers‐Theunissen et al., 2009). Specifically, optimal regeneration of SAM can support DNA methyltransferase activity and temporal expression of critical genes necessary for normal embryonic development (Finnell et al., 2002). A significant association has been found between dietary patterns reflecting one‐carbon metabolism nutrients intake before pregnancy and placental DNA methylation, with solid links found for ‘varied and balanced’ or ‘vegetarian’ patterns and methylation of genes implicated in neurodevelopment and future growth (Lecorguillé et al., 2020; Lecorguillé et al., 2022). Candidate genes linking maternal nutrient exposure to offspring health via one‐carbon metabolism include over 40 different genes and regions of interest (James et al., 2018), including DNMT1, H19, IGF2, LEP, MEG3, NR3C1, PEG3 and RXRA. Interestingly, the biomarkers of one‐carbon metabolism change from preconception across gestation (Gilley et al., 2019), implying a need for a time‐sensitive dietary approach to sustain SAM turnover.

## 3 HIGH‐ENERGY PHOSPHATE METABOLISM

The high‐energy phosphate metabolism produces cellular energy at extremely rapid rates by transferring phosphate groups from adenosine triphosphate and its intermediates, or from stored creatine. Optimising high‐energy phosphagen bioenergetics during periconception might be a major step in normal fetal and neonatal development, particularly for tissues with high or fluctuating energy demands, such as the brain, skeletal muscle or reproductive organs. Creatine is a semi‐essential nutrient that plays a key role in upholding normal phosphagen bioenergetics, with dietary creatine available only in animal‐based foods (Ostojic & Forbes, 2022). Several recent studies demonstrated suboptimal dietary intake of creatine in the general population (Ostojic, 2021), implying the possible risk of inadequate exposure to creatine during periconception. In line with this, creatine deficit could be linked with suboptimal parental fertility (for a detailed review, see Ostojic et al., 2022), while dietary creatine elevates cell energy levels and increases the chance of successful fertilisation (Fakih et al., 1986; Umehara et al., 2018). It is also postulated that a high potential for creatine synthesis may protect the mother from dynamic shifts in the energy required to sustain normal embryonic or fetal development (Moore, 1991). There is an increased requirement for maternal creatine due to the rapid growth and increased metabolic needs of the fetus in the third trimester of pregnancy (Muccini et al., 2021). In a retrospective case‐controlled study, an 18% reduction in maternal serum creatine concentration during the third trimester of pregnancy was associated with a greater incidence of poor perinatal outcomes, which was defined by a composite measure of small for gestational age, preterm birth and admission to neonatal intensive care (Heazell et al., 2012). Several human studies reported reduced creatine concentrations in the brain of preterm infants when compared with term controls at term‐corrected age (Koob et al., 2016). Approximately 6 out of 10 pregnant women consumed dietary creatine below the recommended amounts for an adult female (Ostojic et al., 2021), suggesting a possible risk of creatine malnutrition in this population. Interestingly, maternal creatine intake mitigated neurological injury in the fetal brain and was associated with increased neonatal survival and improved post‐natal growth (Holtzman et al., 1998).

## 4 INFLAMMATION

Maternal chronic inflammation may induce both short‐ and long‐term metabolic reprogramming at several levels, starting from the periconceptional period with effects on the oocyte going through the early stages of embryonic and placental development (Parisi et al., 2021). The systemic inflammation provoked by exposure to lipopolysaccharides during the periconceptional period caused a corticosterone‐independent blunting of the maternal serum proinflammatory cytokine response to innate immune challenge in both male and female offspring (Williams et al., 2011). Interestingly, the suppressed state of neonatal innate immunity was dose‐dependent with respect to the maternal lipopolysaccharide dosage consumed. Various dietary regimens and foods could expose future parents to lipopolysaccharides and concomitant inflammation, including diets low in fish, fresh vegetables, fruits and berries (Ahola et al., 2017) or food supplements (Wassenaar & Zimmermann, 2018). Also, oocytes from overweight/obese women had increased expression of proinflammatory and oxidative stress‐related genes, including chemokine C‐X‐C motif ligand 2 (CXCL2) and dual‐specificity protein phosphatase 1 (DUSP1) (Ruebel et al., 2017). In line with this, a high‐fat diet during the second half of pregnancy appears to increase the expression of fetal liver genes associated with inflammation phenotype, including nuclear factor kappa B 1 (NFKB1), cytokine signalling protein 3 (SOCS3) and DUSP1 (Plata et al., 2014). Interestingly, dietary fructooligosaccharides could ameliorate high‐fat induced intrauterine inflammation and improve lipid profile in the offspring by lowering neutrophil infiltration and decreasing the expression and production of proinflammatory cytokines, such as NFKB1, cyclooxygenase‐2 (COX2), interleukin‐8 and transforming growth factor beta (TGF‐β) (Mohammed et al., 2022). This perhaps extends to other organs, with parental high‐fat, high‐sugar diet can instigate hypothalamic inflammation in offspring, which remained until adulthood (César et al., 2022), implying long‐term metabolic effects of maternal and paternal proinflammatory diet.

## 5 GUT MICROBIOTA

The gut microbiota plays a significant role in human health, with diet being recognised as a critical modulator of gut microbiota diversity and function (Valdes et al., 2018). The current data reveal that gut microbiota may be transmitted from mother to offspring during the birth and its first few days of life through breastfeeding (de Brito Alves et al., 2019). Therefore, maternal gut microbiota‐driven factors, perhaps modulated by diet, could have implications for the etiopathogenesis of various cardiometabolic and neurobehavioral disorders in the next generation. For instance, exposure to endocrine‐disrupting chemicals (EDCs) available from food may disrupt the normal parental gut flora, which may, in turn, result in systemic effects in offspring, with many of the bacteria whose proportions increase with exposure to EDCs in the next generation are associated with inflammatory bowel disease, metabolic disorders and colorectal cancer (Javurek et al., 2016). In addition, maternal periconceptional exposure to antibiotics provokes alterations in offspring behaviour in the absence of maternal infection (Degroote et al., 2016). A preclinical study demonstrated that highly fat‐fed pregnant mice when compared with control‐fed animals were found to be significantly enriched in microbes involved in metabolic pathways favouring fatty acid, ketone, vitamin and bile synthesis (Gohir et al., 2015). Another trial demonstrated that diet‐induced changes in maternal gut microbiota and metabolomic profiles influence the programming of offspring obesity risk in rats (Paul et al., 2016). Also, a paternal high‐protein diet modulates body composition, insulin sensitivity, epigenetics and gut microbiota intergenerationally (Chleilat et al., 2021), with increased abundance of Bifidobacterium, Akkermansia, Bacteroides and Marvinbryantia in high protein‐consumed fathers and/or male and female adult offspring. Still, most studies are focused on associations between maternal gut microbiota and offspring health and thus are limited in identifying direct mechanisms of how the gut microbiota interacts with metabolism in other parts of the body, including fetal metabolism.

## 6 OTHER BIOLOGICAL NETWORKS

Besides one‐carbon metabolism, inflammation and high‐energy phosphate bioenergetics, several other nutrition‐related intracellular signals are recognised as important to support normal periconception, including adiponectin, peroxisome proliferator‐activated receptors (PPARs), proteome‐associated amino acid metabolism, growth‐related protein kinases, fatty acids and sugar. Laudes et al. (2009) recently found that human fetal adiponectin and retinol‐binding protein (RBP)‐4 levels are related to birth weight and maternal obesity, with adiponectin being more likely to have a role in perinatal priming of obesity and insulin resistance than RBP‐4. PPARs are also critical cellular mediators of oocyte quality and ovarian responses to obesity‐induced insulin resistance (Minge et al., 2008), with adipogenic‐regulating genes PPAR gamma (along with vitamin D receptor) could be modulated by maternal diet (Belenchia et al., 2018). Hepatic proteome‐associated amino acid metabolism and antioxidant defence (also energy metabolism) appear to be affected by diet during the pre‐and peri‐conception periods of development (Maloney et al., 2013). In addition, a set of key factors regulating growth and metabolism (such as AMP‐activated protein kinase, phospho‐acetyl CoA carboxykinase, pyruvate dehydrogenase kinase‐4, insulin‐like growth factor [IGF]‐2 receptor, protein kinase C alpha, mechanistic target of rapamycin [mTOR]) are influenced by poor maternal nutrition during periconception (Lie et al., 2013). Interestingly, a recent trial suggested that the placenta could be a true nutrient/resource sensor that orchestrates maternal and fetal signals, with placental IGF‐1 and mTOR recognised as key signalling targets affected by preconception maternal nutrition (Castillo‐Castrejon et al., 2021). In addition, maternal energy intake (especially sugar) appears to be linked with adverse impacts on fetal body composition, not just in diabetics but also in obese mothers and those who consume a high‐sugar diet (as well as a high‐sugar, high‐fat diet), with possible molecular mediators include proinflammatory factors, adipokines and cytokines (Barbour & Hernandez, 2018). Finally, there are many potential effects of fatty acids signalling in the mother that impact fetal development, especially the supplies of long‐chained polyunsaturated fatty acids, linoleic and linolenic acid and their derivatives, arachidonic acid and eicosapentaenoic acid and docosahexaenoic acid (for a detailed review, see Best et al., 2016; and Basak et al., 2021). The cumulative impact of these biological networks on transgenerational health requires an extensive approach by using time‐sensitive nutrigenomics and functional genomics in future studies.

## 7 CONCLUSION

Optimising nutrition during periconception appears to be a relevant target for both the short and long‐term health of the next generation. Diet during this delicate period is often substandard (Hinkle et al., 2020), and this could compromise several key metabolic pathways resulting in poor placental, fetal and neonatal health. One‐carbon metabolism might be a key regulatory network affected by periconceptional nutrition, but other pathways could also be modulated by diet, resulting in the ill health of offspring from the earliest moments in life. Improving our knowledge about how periconceptional nutrition affects next‐generation health might benefit from recognising relevant molecular networks and tackling key modulators by healthy nutrition.

## AUTHOR CONTRIBUTIONS

Sergej M. Ostojic designed and wrote the draft of the manuscript and has primary responsibility for the final content; Laszlo Ratgeber, Jozsef Betlehem and Pongrac Acs revised the manuscript. All authors read and approved the final version of the manuscript.

## CONFLICTS OF INTEREST

Sergej M. Ostojic serves as a member of the Scientific Advisory Board on creatine in health and medicine (AlzChem LLC). Sergej M. Ostojic co‐owns patent “Supplements Based on Liquid Creatine” at European Patent Office (WO2019150323 A1) and has received research support related to nutrition during the past 36 months from the Serbian Ministry of Education, Science, and Technological Development, the Provincial Secretariat for Higher Education and Scientific Research, AlzChem GmbH, ThermoLife International, and Hueston Hennigan LLP. Sergej M. Ostojic is the founder of Centram, a biotechnology startup developing and commercialising innovative nutraceuticals that can support and rejuvenate energy metabolism, the gut‐brain‐muscle axis, and immunity across various health domains. Sergej M. Ostojic does not own stocks and shares in any organisation. The remaining authors declare no conflict of interest.

## Data Availability

This paper was a perspective that did not produce any new data. Accordingly, there is no data to be made available.
